# Comparative effectiveness and safety of vernakalant, flecainide, and amiodarone for atrial fibrillation cardioversion: a propensity score–matched analysis

**DOI:** 10.1186/s12873-026-01604-w

**Published:** 2026-05-07

**Authors:** Giuseppe Dominijanni, Antonio F. Caballero-Bermejo, Álvaro Pineda-Torcuato, Ana Sainz-Herrero, Rosa Capilla-Pueyo, Belén Ruiz-Antorán

**Affiliations:** 1 Emergency Department, Hospital Universitario Puerta de Hierro, C/ Joaquín Rodrigo, 1, Majadahonda, Madrid, 28222 España; 2 Instituto de Investigación Sanitaria Puerta de Hierro Segovia de Arana, Madrid, España; 3 Escuela de Doctorado, Universidad Autónoma de Madrid, Madrid, España; 4 Facultad de Medicina y Ciencias de la Salud, Universidad Nebrija, Madrid, España; 5 Clinical Pharmacology Department, Hospital Universitario Puerta de Hierro, Majadahonda, Madrid, España

**Keywords:** Atrial fibrillation, Vernakalant hydrochloride, Flecainide, Amiodarone, Emergency treatment

## Abstract

**Background:**

Vernakalant is approved in Europe, Canada, and several Asian countries for the pharmacological cardioversion of recent-onset atrial fibrillation (AF), but remains unauthorized in the United States due to FDA safety concerns. Its role in emergency department (ED) management of AF requires further comparative evaluation against other agents.

**Methods:**

We conducted a retrospective observational study including all AF episodes treated with intravenous (iv) vernakalant, flecainide, or amiodarone in a single ED between January 2012 and December 2022. Clinical data were extracted from patient records. The primary outcome was sinus rhythm (SR) conversion during the ED stay. Secondary outcomes included time to SR, ED length of stay, AF recurrence, ED revisits, and rehospitalizations within 6 months, as well as adverse events (AEs) occurring during drug infusion or during the ED stay. Inverse probability of treatment weighting (IPTW) was applied to adjust for baseline confounding. Subgroup analyses explored the impact of demographic and clinical variables on treatment response and safety.

**Results:**

We analyzed 899 AF episodes (vernakalant: 262; flecainide: 151; amiodarone: 486). SR conversion rates were 76.7% (vernakalant), 69.5% (flecainide), and 67.3% (amiodarone). Median time to SR was significantly shorter with vernakalant (0.25 h), compared to flecainide (2.58 h) and amiodarone (8 h; *p* < 0.001). Vernakalant was associated with shorter ED stays. During follow-up, ED revisits and rehospitalizations occurred less frequently in vernakalant-treated patients than in amiodarone-treated patients, although these findings should be interpreted cautiously given baseline differences between groups. AEs occurred in 15.3% (vernakalant), 16.6% (flecainide), and 12.6% (amiodarone); flecainide showed a higher incidence of AEs of special interest. In patients with NYHA class I–II heart failure, vernakalant showed increased efficacy but also higher AE rates.

**Conclusions:**

Vernakalant showed comparable overall efficacy to amiodarone and flecainide for cardioversion of recent-onset AF, with faster SR conversion and shorter ED stays. However, given the retrospective design, non-random treatment allocation, and baseline differences between groups, these comparative findings should be interpreted with caution. Vernakalant may be a useful option in selected patients when rapid cardioversion is desired.

**Supplementary Information:**

The online version contains supplementary material available at 10.1186/s12873-026-01604-w.

## Introduction

Atrial fibrillation (AF) is a supraventricular arrhythmia characterized by chaotic, rapid, and irregular atrial electrical activity, resulting in loss of coordinated atrial contraction and an irregularly irregular ventricular response [1]. In emergency medicine, AF represents the most frequently observed sustained arrhythmia in adults. The clinical relevance of AF lies in its strong link to adverse outcomes such as thromboembolic events, progression to heart failure, ischemic complications, and overall increased mortality [2, 3]. Acute presentations may include palpitations, dyspnea, chest pain, syncope, or hemodynamic instability. The American Heart Association (AHA) emphasizes that the ED is often the first point of diagnosis and management. For stable patients, initial management includes assessment for reversible triggers, rate or rhythm control, and anticoagulation based on thromboembolic risk (e.g., CHA₂DS₂-VASc score) and bleeding risk [4].

Management strategies in the ED range from pharmacological therapy to electrical cardioversion. When cardioversion is performed, short-term observation followed by discharge has been shown to be safe in carefully selected patients [5]. In hemodynamically stable patients, both electrical and pharmacological cardioversion (PCV) are acceptable, safe, and effective for acute rhythm control [6]. In patients with recent-onset atrial fibrillation, PCV to sinus rhythm (SR) is considered one of the most effective treatment strategies—particularly when aiming to avoid the need for sedation associated with electrical cardioversion and to reduce the risk of procedure-related adverse events [7]. Pharmacological options include class IC antiarrhythmics (flecainide, propafenone) and intravenous agents such as amiodarone, ibutilide, or vernakalant [8].

According to European recommendations, flecainide remains a first-line option for patients without structural heart disease, whereas amiodarone is preferred in those with left ventricular dysfunction or heart failure [9]. In recent years, vernakalant has emerged as an attractive alternative in selected patients [8].

Vernakalant, administered intravenously (iv), is approved in Europe, Canada, and several Asian countries for the rapid restoration of sinus rhythm in patients with recent-onset AF (≤ 7 days in non-surgical cases; ≤3 days after cardiac surgery). In contrast, the FDA has withheld approval in the United States, citing safety issues such as hypotension, proarrhythmic events, conduction disturbances, and rare fatal outcomes.

This study seeks to assess, in routine ED practice, the efficacy and safety of intravenous vernakalant in comparison with flecainide and amiodarone for cardioversion of recent-onset AF.

## Methods

### Design and population

We performed a retrospective observational analysis of AF episodes managed in the ED of Hospital Universitario Puerta de Hierro between January 2012 and December 2022. Episodes were eligible if patients received intravenous vernakalant, flecainide, or amiodarone. Because of the retrospective observational design, no study-specific treatment allocation protocol or prospective exclusion criteria were applied beyond receipt of one of these drugs in the ED. Treatment choice reflected routine clinical practice in the emergency department and was influenced by the patient’s clinical profile, underlying structural heart disease, heart failure status, and known contraindications or cautions for each antiarrhythmic agent. In particular, vernakalant was not used in patients with advanced heart failure (NYHA class III–IV), whereas flecainide was generally avoided in patients with structural heart disease, in accordance with routine clinical practice and guideline-based precautions.

The study design and data collection procedures have been described previously [10, 11], and the same protocol was applied here, with the addition of amiodarone-treated cases. The study was approved by the Hospital Universitario Puerta de Hierro’s Research Ethics Committee (REC) (PI 73/22) and conducted in accordance with the Declaration of Helsinki and Good Clinical Practice guidelines. The study was granted a waiver of informed consent by the REC.

### Variables

Demographic and clinical information was extracted from electronic medical records, including prior history, characteristics of the AF episode, laboratory parameters, and outcomes. Symptom onset time was defined according to patient-reported symptom duration at ED presentation. As this was a retrospective real-world study, the true onset of atrial fibrillation before ED arrival could not be objectively verified by continuous ECG monitoring; therefore, this variable should be interpreted as reported symptom onset rather than confirmed arrhythmia onset. The primary endpoint was conversion to sinus rhythm during the ED stay. Secondary outcomes included time to sinus rhythm conversion, ED length of stay, AF recurrence, ED revisits, and rehospitalizations within 6 months. Adverse events were assessed only during antiarrhythmic drug infusion and/or during the ED stay, and no follow-up adverse events were attributed to the index cardioversion drug. Details of variable definitions have been reported in our previous work [10, 11].

### Statistical analysis

Continuous variables were summarized as mean ± standard deviation (SD) or median with interquartile range (IQR), depending on their distribution. Categorical variables were expressed as counts and percentages. Normality was assessed with the Kolmogorov–Smirnov test. Comparisons between groups were performed using Student’s t-test or Mann–Whitney U for continuous variables and chi-square or Fisher’s exact test for categorical variables.

To account for potential confounding, we applied inverse probability of treatment weighting (IPTW) based on propensity scores (PS). Propensity scores were estimated using a multinomial logistic regression model including the following baseline covariates: age, sex, time since symptom onset, history of heart failure, previous episodes of AF, ventricular response at presentation (normal or rapid), and CHA₂DS₂-VASc score. Stabilized weights were computed to create a pseudo-population in which baseline characteristics were balanced across treatment groups. Covariate balance was assessed using standardized mean differences, considering an absolute value < 0.1 as indicative of adequate balance.

The association between treatment group and successful cardioversion was analyzed using both crude and IPTW-adjusted Poisson regression models to estimate relative risks (RRs) with 95% confidence intervals (CIs). Time-to-event outcomes (time to cardioversion and time to AF recurrence) were analyzed using Cox proportional hazards models, and results were reported as hazard ratios (HRs) with 95% CIs.

Subgroup analyses were conducted to explore potential treatment effect modification according to age (< 75 or ≥ 75 years), sex, time from symptom onset (< 12 h or ≥ 12 h), history of heart failure, previous AF, ventricular response, and CHA₂DS₂-VASc score (< 2 or ≥ 2). Interaction terms were tested in the models, and IPTW-adjusted RRs were reported for each subgroup.

All tests were two-sided, and a p-value < 0.05 was considered statistically significant. All statistical analyses were performed using SPSS for Windows version 25.0 (IBM Corp., Armonk, NY, USA) and STATA version 16.1 (StataCorp LLC, College Station, TX, USA).

## Results

### Baseline characteristics and treatment patterns

During the study period, 899 episodes treated with one of the study drugs were identified: 151 episodes were treated with IV flecainide (mean dose 183 mg, SD 74; median 200 mg, IQR 100–200), 262 with IV vernakalant (mean dose 3.78 mg/kg, SD 0.98; median 3 mg/kg, IQR 3–5), and 486 received IV amiodarone (mean total dose 1062 mg, SD 298; median 1200 mg, IQR 1200–1200). Table 1 summarizes the demographic and clinical features of patients across the three treatment groups, both before and after IPTW adjustment. A propensity score (PS) was calculated to estimate each patient’s probability of receiving each treatment based on baseline characteristics and to reduce selection bias. Regarding episode-specific characteristics, the three treatment groups showed overall comparability after IPTW adjustment. The proportion of first AF episodes was 29.2% in the vernakalant group, 24.2% in the amiodarone group, and 21.4% in the flecainide group, with no statistically significant differences between groups (*p* = 0.159).

**Table 1:** Baseline characteristics of patients according to antiarrhythmic treatment group

	Raw analysis							IPTW analysis						
	Vernakalant (*N* = 262)	Amiodarone (*N* = 486)	Flecainide (*N* = 151)	*p* value	Standardized difference (%)			Vernakalant (*N* = 267)	Amiodarone (*N* = 484)	Flecainide (*N* = 145)	Standardized difference (%)			
					Vernakalant vs Amiodarone	Vernakalant vs Flecainide	Amiodarone vs Flecainide				Vernakalant vs Amiodarone	Vernakalant vs Flecainide	Amiodarone vs Flecainide	
Sex, male, n (%)	168 (64,1)	231 (47,5)	69 (54,7)	< 0,001	3,390	3,763	0,361	156 (58,4)	246 (50,8)	55 (37,9)	1,531	4,192	2,619	
Age, years, mean (SD)	60,7 (12,7)	67,9 (12,8)	61,4 (12,5)	< 0,001	0,565	0,054	0,500	64,0 (13,5)	64,8 (13,5)	63,1 (12,1)	2,574	2,428	0,144	
Underlying medical conditions, n (%)											1,078	0,720	1,795	
Hypertension	111 (42,4)	327 (67,3)	89 (58,9)	< 0,001	5,168	3,346	1,747	130 (48,7)	297 (61,4)	88 (60,7)	1,564	0,787	2,357	
Diabetes	23 (8,8)	91 (18,7)	14 (9,3)	< 0,001	2,905	0,174	2,734	34 (12,7)	80 (16,5)	15 (10,3)	0,991	0,649	1,629	
Dyslipidemia	85 (32,4)	227 (46,7)	46 (30,5)	< 0,001	2,956	0,409	3,375	93 (35,0)	206 (42,6)	45 (31,3)	1,331	6,783	5,658	
Cerebrovascular accident	12 (4,6)	43 (8,8)	8 (5,3)	0,062	1,686	0,323	1,370	15 (5,6)	39 (8,1)	6 (4,1)	2,574	2,428	0,144	
Acute coronary syndrome	39 (14,9)	76 (15,6)	1 (0,7)	< 0,001	0,195	5,491	5,660	50 (18,7)	67 (13,8)	0	1,078	0,720	1,795	
Heart failure														
No heart failure	248 (94,7)	398 (81,9)	146 (96,7)	< 0,001	4,064	0,987	4,931	252 (94,4)	401 (82,7)	139 (95,9)	3,478	0,699	4,116	
Mild (NYHA I–II)	14 (5,3)	71 (14,6)	5 (3,3)	< 0,001	3,145	0,987	4,038	15 (5,6)	67 (13,8)	6 (4,1)	2,798	0,699	3,448	
Moderate (NYHA III)	0 (0,00)	17 (3,5)	0 (0,00)	0,001	2,693	-	2,693	0 (0)	17 (3,5)	0 (0)	2,693	-	2,693	
Cardiomyopathy	4 (1,5)	42 (8,6)	2 (1,3)	< 0,001	3,286	0,170	3,414	3 (1,1)	36 (7,4)	3 (2,1)	3,162	0,798	2,511	
Other prior arrhythmias	89 (34,0)	70 (14,4)	19 (12,6)	< 0,001	4,701	5,233	0,527	109 (40,8)	87 (18,0)	11 (7,6)	5,169	8,409	3,151	
Structural cardiomyopathy	13 (5,0)	77 (15,8)	2 (1,3)	< 0,001	3,595	2,130	5,369	12 (4,5)	80 (16,5)	5 (3,4)	3,992	0,565	4,485	
CHA₂DS₂-VASc > = 2	104 (40,9)	332 (68,3)	70 (46,4)	< 0,001	5,724	1,111	4,541	134 (51,3)	294 (60,7)	77 (46,5)	1,902	0,961	2,877	
Previous antiarrhythmic, n (%)														
Amiodarone	8 (3,1)	156 (32,1)	2 (1,3)	< 0,001	8,235	1,229	9,067	9 (3,4)	136 (28,1)	0 (0)	7,208	2,653	8,841	
Beta-blocker	105 (40,1)	202 (41,6)	68 (45,0)	0,613	0,305	0,992	0,687	122 (45,7)	212 (43,8)	64 (44,1)	0,382	0,322	0,060	
Calcium channel blockers	19 (7,3)	77 (15,8)	9 (6,0)	< 0,001	2,683	0,522	3,184	23 (8,6)	68 (14,0)	8 (5,0)	1,712	1,434	3,106	
Flecainide	13 (5,0)	11 (2,3)	90 (59,6)	< 0,001	1,444	14,381	15,794	36 (13,5)	58 (12,0)	19 (13,1)	0,450	0,118	0,332	
Propafenone	5 (1,9)	0 (0)	1 (0,7)	0,009	1,968	1,061	1,187	4 (1,5)	0 (0)	2 (1,4)	1,745	0,084	1,685	
Dronedarone	3 (1,1)	15 (3,1)	0 (0)	0,031	1,398	1,491	2,529	3 (1,1)	13 (2,7)	0 (0)	1,174	1,491	2,356	
Other	1 (0,4)	1 (0,2)	0 (0)	0,725	0,366	0,896	0,633	1 (0,4)	2 (0,4)	0 (0)	0,000	0,896	0,896	
Anticoagulants, n (%)	98 (37,4)	312 (64,2)	86 (57,0)	< 0,001	5,564	4,004	1,478	126 (47,2)	302 (62,4)	82 (56,6)	3,090	1,890	1,184	
Patients with previous episodes of FA, n (%)	177 (67,6)	360 (74,1)	131 (86,8)	< 0,001	1,434	4,642	3,185	188 (70,4)	367 (75,8)	114 (78,6)	1,220	1,890	0,668	
Number of previous episodes, mean (SD)	1,97 (2,57)	2,02 (2,88)	1,99 (3,82)	0,969	0,018	0,006	0,009	2,2 (2,8)	2,1 (2,8)	1,6 (2,3)	0,059	0,070	0,132	
Time from symptom onset to treatment administration, min, mean (SD)	11,11 (20,22)	15,82 (25,05)	10,43 (14,34)	0,004	0,207	0,039	0,264	17,8 (34,3)	13,7 (21,6)	15,5 (23,9)	0,036	0,234	0,195	

Paroxysmal AF was the predominant pattern across all groups (70.8% with vernakalant, 75.8% with amiodarone, and 78.6% with flecainide). A rapid ventricular rate (> 110 bpm) was observed in 61.4% of vernakalant-treated episodes, 70.7% with amiodarone, and 72.4% with flecainide. Palpitations were the most frequent symptom (81.6%–83.4%, *p* = 0.409), while dyspnea (23.8%) and chest pain (23.8%) were more common in the amiodarone group (*p* = 0.003 and *p* = 0.119, respectively). Regarding the administration regimen, flecainide was mainly used as a single bolus in 87.6% of episodes, while amiodarone was almost exclusively administered as bolus followed by infusion (84.1%). Vernakalant showed a more balanced distribution between single bolus (55.1%) and bolus plus infusion (44.9%) (*p* < 0.001).

### Efficacy outcomes

**Fig. 1:**
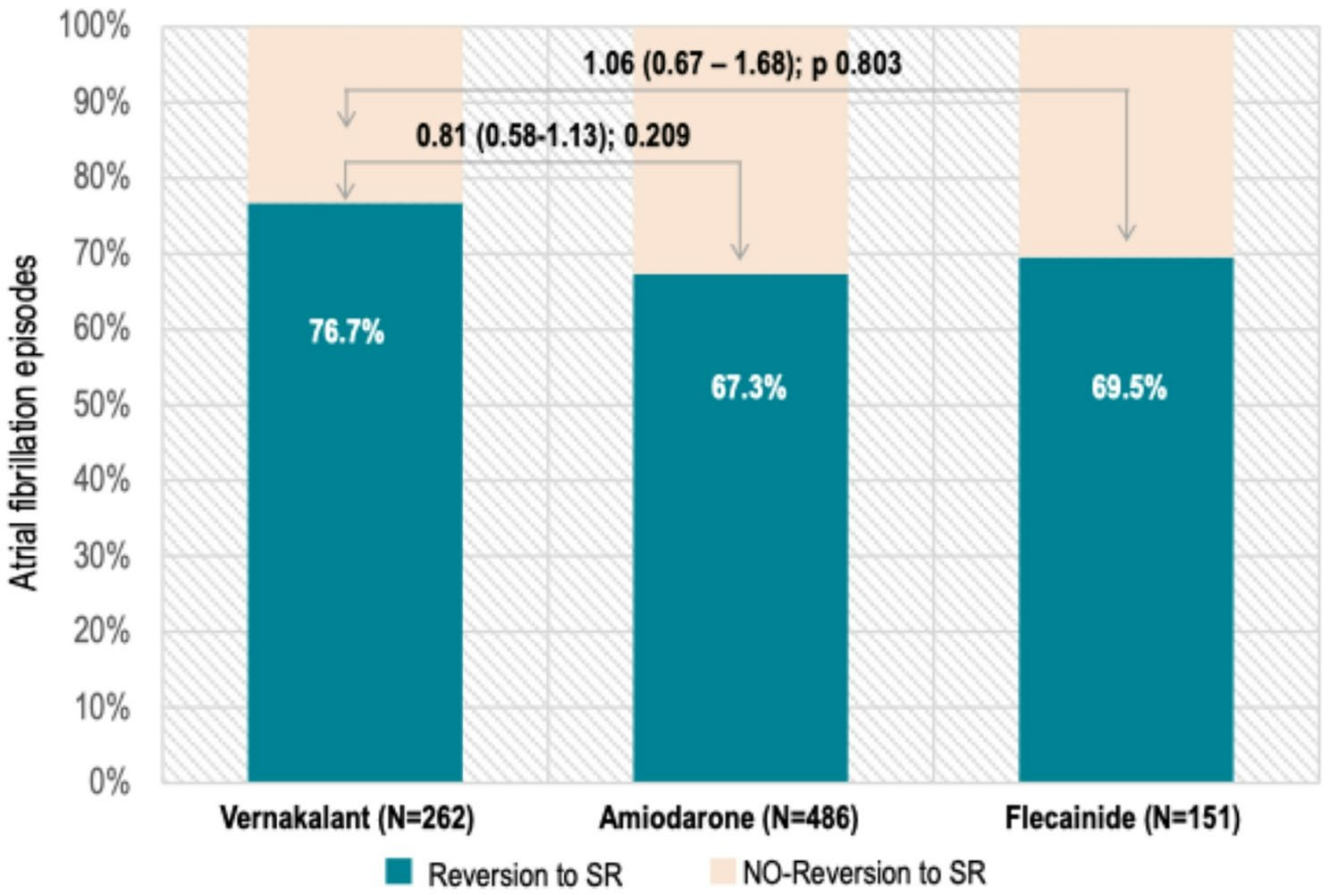
Effectiveness: reversion to SR. Total episodes. wRR (95% CI); p

Conversion to sinus rhythm during the ED stay occurred in 76.7% of episodes managed with vernakalant, 69.5% with flecainide, and 67.3% with amiodarone. After IPTW adjustment, no statistically significant differences in successful reversion rates were observed between vernakalant and amiodarone (RR 0.81, 95% CI: 0.58–1.13; *p* = 0.209), or between vernakalant and flecainide (RR 1.06, 95% CI: 0.67–1.68; *p* = 0.803) (Fig. 1).

**Fig. 2:**
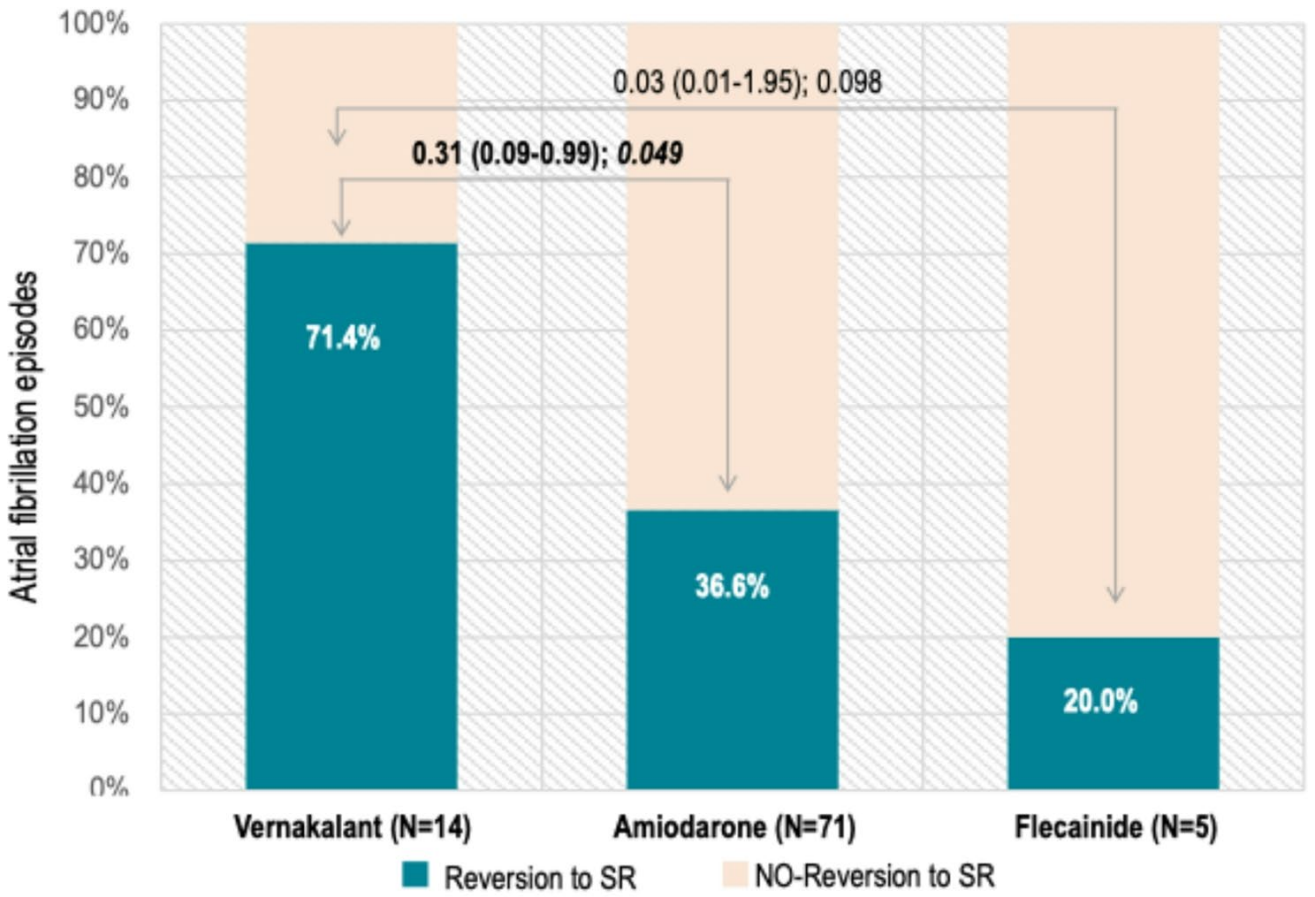
Effectiveness: reversion to SR. Subgroup of patients with heart failure (NYHA class I–II). wRR (95% CI); p

Subgroup analyses revealed that vernakalant was significantly more effective than amiodarone specifically in patients with a history of heart failure (NYHA class I–II), achieving successful cardioversion in 71.4% of cases (10/14) compared to 36.6% with amiodarone (26/71) (RR 0.31, 95% CI: 0.09–0.99; *p* = 0.049) (Table 2; Fig. 2). No other significant differences in effectiveness were observed between vernakalant and the other treatments across subgroups defined by age, sex, time since symptom onset, prior history of AF, AF type (first episode or paroxysmal), ventricular response, or CHA₂DS₂-VASc score (Table 2).

**Table 2:** Subgroup analysis: conversion to Sinus Rhythm (SR) showing the weighted RR from IPTW analysis

	Vernakalant	Amiodarone	RR IPTW (95% CI); *p*	Flecainide	RR IPTW (95% CI); *p*
	*n*/*N* (%)	*n*/*N* (%)		*n*/*N* (%)	
**Total**	**201/262 (76.7)**	**327/486 (67.3)**	**0.81 (0.58–1.13); 0.209**	**105/151 (69.5)**	**1.06 (0.67–1.68); 0.803**
Age					
<75 years	169/208 (77.5)	216/320 (67.5)	0.70 (0.47–1.04); 0.078	91/127 (71.7)	1.32 (0.75–2.33); 0.326
* ≥*75 years	32/44 (72.7)	111/166 (66.9)	1.15 (0.62–2.11); 0.663	14/24 (58.3)	0.51 (0.21–1.21); 0.129
Sex					
Male	130/168 (77.4)	164/231 (71.0)	0.72 (0.45–1.16); 0.176	51/69 (73.9)	1.33 (0.60–2.96); 0.483
Female	71/94 (75.5)	163/255 (63.9)	0.98 (0.61–1.57); 0.928	54/82 (65.9)	1.19 (0.66–2.14); 0.567
Time since symptom onset					
<12 h	169/207 (81.6)	250/329 (76.0)	0.73 (0.47–1.13); 0.157	83/119 (69.7)	0.79 (0.44–1.41); 0.430
* ≥*12 h	32/55 (58.2)	77/157 (49.0)	1.00 (0.57–1.78); 0.988	22/32 (68.8)	1.70 (0.75–3.87); 0.204
History of heart failure (HF)					
Yes	10/14 (71.4)	31/88 (35.2)	0.31 (0.09–0.97); 0.045	1/5 (20.0)	0.026 (0.001–1.95); 0.098
No	191/248 (77.0)	296/398 (74.4)	1.11 (0.78–1.60);0.544	104/146 (71.2)	1.19 (0.74–1.92); 0.473
Previous AF episodes					
Yes	129/177 (72.9)	229/360 (73.6)	0.93 (0.64–1.35); 0.712	85/131 (64.9)	0.98 (0.60–1.60); 0.922
No	72/85 (84.7)	98/126 (77.8)	0.55 (0.26–1.17); 0.119	20/20 (100)	-
Type of AF					
First episode	73/86 (84.9)	98/126 (77.8)	0.56 (0.26–1.19); 0.129	20/20 (100)	-
Paroxysmal	128/176 (72.7)	229/360 (63.6)	0.92 (0.64–1.34); 0.677	85/131 (64.9)	0.97 (0.59–1.59); 0.895
Ventricular response					
Normal	69/96 (71.9)	77/134 (57.5)	0.88 (0.51–1.51); 0.647	35/46 (76.1)	2.01 (0.82–4.90); 0.123
Rapid	132/166 (79.5)	250/353 (71.0)	0.74 (0.48–1.13); 0.159	70/105 (66.7)	0.79 (0.46–1.37); 0.406
CHA₂DS₂-VASc Score					
< 2	122/150 (81.3)	111/154 (72.1)	0.61 (0.35–1.07); 0.084	61/81 (65.3)	0.75 (0.37–1.52); 0.425
*≥* 2	72/104 (69.2)	216/332 (65.1)	1.08 (0.71–1.65); 0.711	44/70 (62.9)	1.32 (0.71–2.46); 0.379

**Fig. 3:**
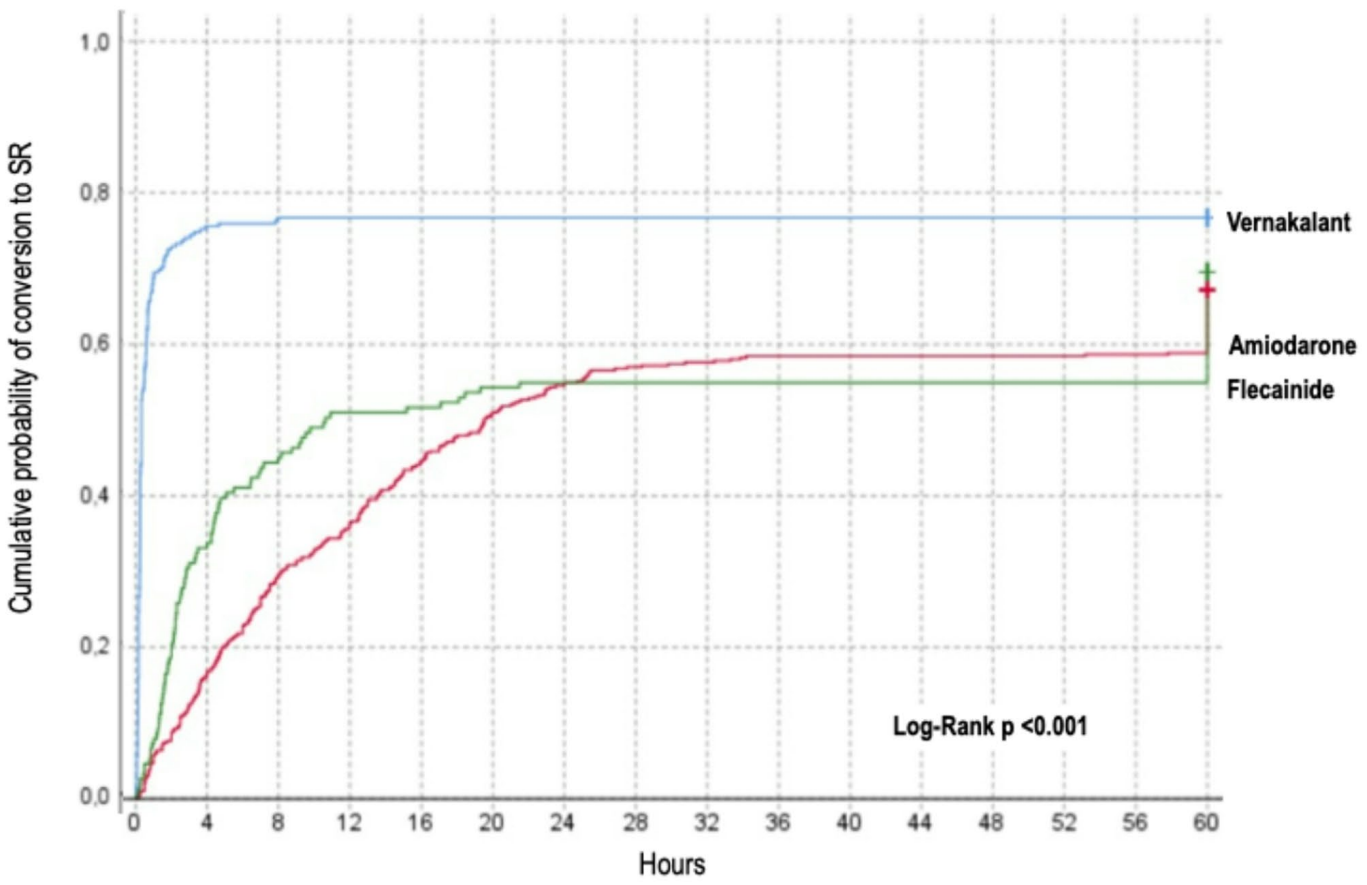
Time to conversion to sinus rhythm by treatment group (Kaplan-Meier analysis)

Median time from drug administration to SR reversion was significantly shorter with vernakalant [0.25 h, IQR: 0.17–0.58] compared to amiodarone [8 h, IQR: 3.58–15.91; HR 0.06, 95% CI: 0.04–0.09; *p* < 0.001] and flecainide [2.58 h, IQR: 1.52–6.42; HR 0.20, 95% CI: 0.14–0.28; *p* < 0.001] (Fig. 3). ED stay was also significantly shorter for vernakalant-treated episodes [4.25 h, IQR: 3.08–7.23] compared to amiodarone [21.43 h, IQR: 10.4–32.73; IPTW-adjusted mean difference: 28.01 h, 95% CI: 18.75–37.27; *p* < 0.001], but no significant difference was observed between vernakalant and flecainide [10.25 h, IQR: 5.4–16.27; mean difference: 0.43 h, 95% CI: − 5.81 to 6.68; *p* = 0.892].

**Fig. 4:**
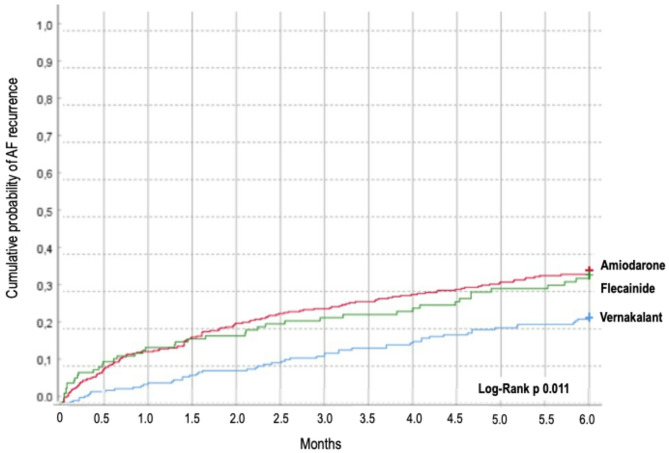
Cumulative probability of atrial fibrillation recurrence during 6-month follow-up, by treatment group

Regarding recurrence during the first 6 months after the index AF episode, the median time to AF recurrence was longer in the vernakalant group [2.45 months, IQR: 0.98–4.1] compared to amiodarone [1.41 months, IQR: 0.42–3.08; mean difference: − 0.034 months, 95% CI: − 0.066 to − 0.03; *p* = 0.034], with no significant difference observed between vernakalant and flecainide [1.30 months, IQR: 0.20–3.95; mean difference: 0.07 months, 95% CI: − 0.04 to 0.51; *p* = 0.765] (Fig. 4).

During 6-month follow-up, ED revisits and hospitalizations occurred less frequently among vernakalant-treated patients than among amiodarone-treated patients [IPTW-adjusted mean difference: 0.19, 95% CI: 0.07–0.31; *p* = 0.002], whereas differences versus flecainide were not significant [mean difference: − 0.01, 95% CI: − 0.02 to 0.12; *p* = 0.788]. Rehospitalizations were also less frequent in the vernakalant group than in the amiodarone and flecainide groups [vs amiodarone: mean difference: 0.14, 95% CI: 0.09–0.19; *p* < 0.001; vs. flecainide: mean difference: 0.15, 95% CI: 0.04–0.26; *p* = 0.007]. These follow-up outcomes should be interpreted cautiously (Table 3).

**Table 3:** Analysis of secondary variables showing the weighted

	Vernakalant	Amiodarone	RR/HR IPTW (IC 95%); *p*	Mean Difference IPTW (95% CI)	Flecainide	RR/HR IPTW (IC 95%); *p*	Mean Difference IPTW (95% CI)
Time from drug administration to reversion (hours), median (IQR)	0.25 (0.17–0.58)	8 (3.58–15.91)	HR 0.06 (0.04–0.09); <0.001	-	2.58 (1.52–6.42)	HR 0.197 (0.14–0.28); <0.001	-
Stay in ED (hours), median (IQR)	4.25 (3.08–7.23)	21.43 (10.4-32.73)		28.01 ( 18.75–37.27); <0.001	10.25 (5.4-16.27)		0.43 (-5.81-6.68); 0.892
Time to recurrence (months), median (IQR)	2.45 (0.98–4.1)	1.41 (0.42–3.08)		-0.034 (-0.066- -0.03); 0.034	1.30 (0.20–3.95)		0.07 (-0.04–0.51);0.765
AF recurrence at 6 months, n/N (%)	54/262 (20.6)	146/484 (30.0)	RR 1.35 (0.95–1.91); 0.095		44/151 (29.1)	RR 0.65 (0.38–1.10); 0.110	
Number of ED revisits at 6 months, mean (SD)	0.24 (0.53)	0.51 (0.99)		0.19 (0.07–0.31); 0.002	0.48 (0.88)		-0.01 (-0.02-0.12); 0.788
Number of rehospitalizations at 6 months, mean (SD)	0.01 (0.08)	0.16 (0.42)		0.14 (0.09–0.19); <0.001	0.18 (0.42)		0.15 (0.04–0.26); 0.007

Mean Difference, RR or HR from IPTW analysis

### Safety outcomes

Adverse events (AEs) occurred in 15.3% of vernakalant episodes, 12.6% of amiodarone, and 16.6% of flecainide. The IPTW-adjusted risk of experiencing any AE was not significantly different between vernakalant and amiodarone [RR 0.67, 95% CI: 0.44–1.03; *p* = 0.066], or between vernakalant and flecainide [RR 1.12, 95% CI: 0.66–1.90; *p* = 0.660].

Adverse events of special interest—including hypotension, bradycardia, complete atrioventricular block, and ventricular tachycardia—occurred in 3.1% of patients treated with vernakalant, 9.5% with amiodarone, and 13.2% with flecainide. The incidence of these events was significantly higher in the flecainide group compared to vernakalant [RR 2.94, 95% CI: 1.45–5.99; *p* = 0.003], while the difference between vernakalant and amiodarone was not statistically significant [RR 1.68, 95% CI: 0.90–3.12; *p* = 0.104] (Table 4).

**Table 4:** Adverse events by treatment group with IPTW-adjusted relative risks

	Vernakalant	Amiodarone	RR IPTW (IC 95%); *p*	Flecainide	RR IPTW (IC 95%); *p*
	*n* = 262 *N* (%)	*n* = 486 *N* (%)		*n* = 151 *N* (%)	
**Total**	**40 (15.3)**	**61 (12.6)**	**0.67 (0.44–1.03); 0.066**	**25 (16.6)**	**1.12 (0.66–1.90); 0.660**
AEs of special interest*	**8 (3.1)**	**46 (9.5)**	**1.68 (0.90–3.12); 0.104**	**20 (3.2)**	**2.94 (1.45–5.99); 0.003**
Dysgeusia	4 (1.50)	0 (0.00)		0 (0.00)	
General malaise/Sweating	7 (2.70)	12 (2.50)		4 (2.60)	
Dizziness	4 (1.50)	16 (3.30)		10 (6.60)	
Generalized pruritus	3 (1.10)	2 (0.40)		0 (0.00)	
Dyspnea	2 (0.80)	5 (1.00)		0 (0.00)	
Cough	2 (0.80)	0 (0.00)		0 (0.00)	
Paresthesia	1 (0.40)	0 (0.00)		0 (0.00)	
Nausea and vomiting	9 (3.40)	11 (2.30)		2 (1.30)	
Hypotension (SBP < 90 mmHg)	2 (0.80)	31 (6.40)		16 (10.60)	
Bradycardia	4 (1.50)	24 (4.90)		12 (7.90)	
Atrial flutter	11 (4.20)	2 (0.40)		4 (2.60)	
Complete AV block	0 (0.00)	0 (0.00)		1 (0.70)	
Ventricular tachycardia	2 (0.80)	0 (0.00)		1 (0.70)	
Arthralgias	1 (0.40)	2 (0.40)		0 (0.00)	
Throat itchiness	3 (1.10)	0 (0.00)		0 (0.00)	
Others	5 (1.90)	1 (0.20)		0 (0.00)	

* Hypotension, bradycardia, complete AV block y VT

Subgroup analysis revealed that female patients had fewer AEs with vernakalant than with amiodarone [RR 0.54, 95% CI: 0.31–0.95; *p* = 0.032]. Additionally, among patients with normal ventricular response, the incidence of AEs was lower with vernakalant compared to amiodarone [RR 0.32, 95% CI: 0.14–0.72; *p* = 0.005]. In contrast, in patients with heart failure classified as NYHA I–II, adverse events were significantly more frequent in those treated with vernakalant (42.9%) compared to amiodarone (8.5%) [RR 0.08, 95% CI: 0.02–0.33; *p* < 0.001], indicating a higher risk of AEs in this subgroup. No significant differences were observed across other subgroups, including age, CHA₂DS₂-VASc score, or rapid ventricular response (Table 5).

**Table 5:** Adverse event risk by subgroup (IPTW analysis)

*n*/*N* (%)	Vernakalant	Amiodarone	RR IPTW (IC 95%); *p*	Flecainide	RR IPTW (IC 95%); *p*
**Total**	**40/262 (15.30)**	**61/486 (12.6)**		**25/151 (16.6)**	
Age					
<75 years	33/218 (15.1)	29/320 (9.1)	0.55 (0.32–0.93); 0.025	19/127 (15.0)	1.25 (0.68–2.28); 0.472
* ≥*75 years	7/44 (15.9)	32/166 (19.3)	0.99 (0.48–2.07); 0.995	6/24 (25.0)	0.84 (0.28–2.55); 0.758
Sex					
Male	20/168 (11.9)	25/231 (10.8)	0.78 (0.41–1.51); 0.465	11/69 (15.9)	1.79 (0.77–4.12); 0.173
Female	20/94 (21.3)	36/255 (14.1)	0.54 (0.31–0.95); 0.032	14/82 (17.1)	0.69 (0.35–1.38); 0.303
History of heart failure (HF)					
No	34/248 (13.7)	50/398 (12.6)	0.78 (0.49–1.23); 0.291	23/146 (15.8)	1.19 (0.68–2.08); 0.533
NYHA I-II	6/14 (42.90)	6/71 (8.5)	0.08 (0.02–0.33); <0.001	2/5 (40.0)	0.97 (0.14–6.86); 0.972
NYHA III-IV	-	5/17 (29.4)	-	-	-
Ventricular rate					
Normal	16/96 (16.7)	13/134 (9.7)	0.32 (0.14–0.72); 0.005	8/46 (17.4)	0.89 (0.35–2.30); 0.820
Rapid	24/166 (14.5)	48/352 (13.6)	0.91 (0.54–1.54); 0.720	17/105 (16.2)	1.29 (0.68–2.48); 0.433
CHA₂DS₂-VASc Score					
< 2	20/150 (13.30)	13/154 (8.40)	0.58 (0.28–1.20); 0.143	12/81 (14.8)	1.16 (0.52–2.58); 0.718
*≥* 2	19/104 (18.3)	48/332 (14.5)	0.66 (0.39–1.12); 0.126	13/70 (18.6)	1.13 (0.55–2.29); 0.736

## Discussion

Our findings suggest that iv vernakalant may be as effective as amiodarone and flecainide for pharmacological cardioversion of recent-onset AF in the ED setting, although these comparisons should be interpreted with caution given the retrospective design and the incomplete comparability between treatment groups. It should be clarified that the episodes included in this study correspond to recent-onset AF evaluated and treated in the ED, regardless of whether symptom onset occurred at home or after hospital arrival. In addition, patient-reported symptom onset may not accurately reflect the true onset of atrial fibrillation. Consequently, the actual duration of AF before ED arrival may have varied across patients and may have influenced pharmacological cardioversion efficacy. This limitation is inherent to retrospective ED-based studies in which continuous rhythm monitoring before presentation is unavailable.

Although most episodes were classified as paroxysmal AF, pharmacological cardioversion was initiated because spontaneous reversion had not occurred at the time of ED evaluation. While early treatment and the paroxysmal nature of AF may have contributed to high overall conversion rates, this potential effect applies similarly across treatment groups and does not fully explain the marked differences observed in time to cardioversion and ED length of stay. Although no significant differences in overall conversion rates were observed after adjustment for baseline differences using IPTW, compared with amiodarone and flecainide, vernakalant restored sinus rhythm in a considerably shorter time frame, which translated into reduced ED lengths of stay.

These findings are supported by real-world data collected in an ED recognized for its expertise in AF management and its function as a regional referral center.

Subgroup analyses revealed that vernakalant was significantly more effective than amiodarone in patients with a history of heart failure. Although comparative evidence in this population is limited, vernakalant has demonstrated efficacy and safety in experimental models of heart failure without inducing proarrhythmia [12]. Amiodarone, on the other hand, is widely used in the management of heart failure and has been shown to improve ejection fraction, although its overall clinical benefit remains variable and requires close monitoring due to potential toxicit [13]. Our findings contribute additional evidence, suggesting that in patients with NYHA class I–II heart failure (given that vernakalant is contraindicated in NYHA III–IV), vernakalant may offer greater effectiveness. However, safety analyses also showed that adverse events were significantly more frequent in this subgroup when treated with vernakalant (42.9%) compared to amiodarone (8.5%), indicating a higher risk of complications. These results are consistent with previous studies [10, 11] and support the preferential use of amiodarone in patients with heart failure, regardless of NYHA classification.

Our results are consistent with previous studies showing a reduction in time to conversion to sinus rhythm [14, 15]. In our study, the median time from drug administration to SR reversion was significantly shorter with vernakalant compared to amiodarone and flecainide. ED stay was also significantly shorter for vernakalant-treated episodes compared to amiodarone, but no significant difference was observed between vernakalant and flecainide. Several clinical and methodological factors may influence the apparent efficacy of pharmacological cardioversion and should be considered when interpreting and comparing results across studies. These include the overall atrial fibrillation burden and duration of the index episode, prior exposure to antiarrhythmic drugs, history of previous cardioversions, type and severity of underlying structural heart disease and other comorbidities, as well as differences in monitoring strategies during the emergency department stay (continuous versus intermittent ECG monitoring). Many of these variables are heterogeneously reported or not systematically captured in real-world studies, including ours, and may partially account for variability in efficacy rates across different clinical settings.

Our findings suggest that vernakalant may be a useful option for selected patients with recent-onset AF in the emergency setting, particularly when rapid cardioversion is clinically desirable. The rapid onset observed with vernakalant may be of interest in time-sensitive ED settings, where shorter time to cardioversion could be operationally relevant. This quicker response was associated with significantly shorter ED stays compared with amiodarone, which may be relevant in time-sensitive emergency care settings. Given the high rate of successful cardioversion observed with vernakalant, its use may be relevant for patient flow in selected emergency care settings, although this should be interpreted cautiously within the limitations of an observational study.

Moreover, as hospital care remains a major contributor to AF-related healthcare costs in Europe, the shorter ED stay observed with vernakalant may have potential implications for resource use, although this should not be interpreted as a direct demonstration of cost-effectiveness in our study [16].

During 6-month follow-up, ED revisits occurred less frequently in vernakalant-treated patients than in amiodarone-treated patients, whereas no significant differences were observed versus flecainide. Rehospitalizations also occurred less frequently in the vernakalant group than in the amiodarone and flecainide groups. However, these medium-term outcomes are unlikely to be causally attributable to a single short infusion of an antiarrhythmic drug administered for acute cardioversion. Although IPTW adjustment was used to improve comparability across treatment groups, residual confounding remains likely for 6-month follow-up outcomes. Rather, these events are more plausibly related to baseline comorbidities, underlying structural heart disease, functional status, and subsequent long-term management. In our cohort, patients treated with amiodarone had a higher baseline risk profile, which may have contributed to greater downstream healthcare utilization. Because revisits and rehospitalizations were not systematically adjudicated as AF-related or unrelated, these findings should be interpreted as descriptive and hypothesis-generating only, rather than as evidence of a sustained treatment effect of the index cardioversion drug.

One of the most debated aspects of vernakalant use is its safety profile, which notably led the FDA to reject its marketing application due to concerns about severe hypotension, ventricular arrhythmias, conduction abnormalities, and even death. All adverse events reported in this study occurred during the antiarrhythmic drug infusion or the emergency department stay and were directly related to the acute cardioversion process; no long-term or follow-up adverse events were attributed to the index cardioversion drug. In our study, however, overall tolerability was comparable across the three agents, with no group showing an excess burden of adverse events. AEs were reported in 15.3% of episodes treated with vernakalant, 12.6% with amiodarone, and 16.6% with flecainide. After IPTW adjustment, the risk of experiencing any AE did not differ significantly between vernakalant and either amiodarone or flecainide.

AEs of special interest—namely hypotension, bradycardia, complete atrioventricular block, and ventricular tachycardia—occurred in 3.1% of patients receiving vernakalant, compared to 9.5% with amiodarone and 13.2% with flecainide. Events such as hypotension and bradycardia occurred less frequently in patients receiving vernakalant than in those treated with flecainide, whereas rates were broadly similar when contrasted with amiodarone. These results align with previous reports, which have not detected any clinically significant safety issues with vernakalant under routine clinical practice [17–21].

Subgroup analyses revealed that female patients experienced fewer AEs with vernakalant than with amiodarone. These findings are consistent with previous studies [22], which have shown that women treated with amiodarone have a significantly higher risk of developing bradyarrhythmias requiring permanent pacemaker implantation compared to men—even after adjusting for dose, body weight, and comorbidities. This increased risk in women appears to be independent of heart failure status and is likely related to sex-based differences in pharmacokinetics and pharmacodynamics, as well as a generally higher female susceptibility to adverse effects from antiarrhythmic drugs [23]. In contrast, for vernakalant, no clear sex-related differences in safety profiles have been reported among patients with AF and heart failure. Similarly, among patients with normal ventricular response, vernakalant was associated with a lower incidence of AEs compared to amiodarone. No significant differences in safety outcomes were found across other subgroups, including age, CHA₂DS₂-VASc score, or rapid ventricular response.

This study has certain limitations. The retrospective observational nature precludes firm causal inferences, and although IPTW adjustment was applied, the possibility of residual confounding cannot be entirely excluded. Importantly, patients receiving amiodarone were more clinically compromised at baseline (Table 1), likely reflecting treatment selection in routine practice. Although IPTW adjustment was applied, residual confounding related to baseline disease severity cannot be fully excluded. Because the information was obtained from medical records rather than collected prospectively, errors in classification and unmeasured variables may have influenced the results.

In addition, treatment allocation was not randomized or standardized; decisions were made individually by the treating physicians, which may have introduced variability in outcomes. Infusion regimens were also heterogeneous and reflected routine clinical practice rather than protocol-mandated administration schedules. Therefore, the dosing strategies used for flecainide, amiodarone, and vernakalant were not always fully aligned with previously published protocols, which may also have influenced both efficacy and adverse event rates. Finally, in our center intravenous amiodarone has historically been the predominant first-line therapy for pharmacological cardioversion, independent of structural heart disease. This institutional practice may have biased drug selection, with flecainide or vernakalant more often prescribed to younger and clinically less complex patients, thereby potentially contributing to selection bias [1]. Furthermore, ED revisits and rehospitalizations during the 6-month follow-up period could not be systematically adjudicated as AF-related or unrelated to AF, nor as related or unrelated to underlying structural heart disease, due to the retrospective nature of the data collection. This limits the interpretability of these outcomes and further supports their treatment as descriptive and hypothesis-generating findings only.

The novelty of this study lies in the real-world comparison of vernakalant, amiodarone, and flecainide for pharmacological cardioversion of recent-onset AF in a high-volume ED setting. Unlike prior studies focusing predominantly on selected trial populations, our analysis reflects contemporary routine clinical practice and incorporates adjustment for baseline differences using inverse probability of treatment weighting. Importantly, our findings emphasize clinically relevant process-of-care outcomes, particularly time to SR restoration and ED length of stay, which are highly relevant in resource-constrained emergency settings and have not been consistently addressed in previous comparative studies.

Overall, our findings suggest that vernakalant may be a useful option in selected patients with recent-onset atrial fibrillation, particularly when rapid pharmacological cardioversion is desired. Its benefit may be particularly relevant in selected patients without advanced heart failure, although caution is warranted in patients with NYHA class I–II heart failure given the higher adverse event rates observed in this subgroup. Vernakalant may be a useful option in selected ED settings or in cases where other antiarrhythmic agents are less suitable.

## Conclusion

In this real-world analysis, iv vernakalant showed comparable overall efficacy to amiodarone and flecainide for cardioversion of recent-onset AF, while achieving faster restoration of sinus rhythm and shorter ED stays. However, given the retrospective design, non-random treatment allocation, residual baseline differences between groups, and heterogeneous infusion protocols, these comparative findings should be interpreted with caution. Vernakalant may represent a useful option in selected patients with recent-onset AF, particularly when rapid pharmacological cardioversion is clinically desirable.

## Supplementary Information

Below is the link to the electronic supplementary material.

Supplementary Material 1

## Data Availability

The datasets used and/or analysed during the current study are available from the corresponding author on reasonable request.

## Abbreviations

AF: Atrial fibrillation
ED: Emergency department
AHA: American heart association
PCV: Pharmacological cardioversion
iv: Intravenous
FDA: Food and drug administration
SR: Sinus rhythm
REC: Research ethics committee
SD: Standard deviation
IQR: Interquartile ranges
IPTW: Inverse probability of treatment weighting
PS: Propensity score
RR: Relative risk
CI: Confidence interval
HR: Hazard ratio
NYHA: New York heart association
AE: Adverse event
SMD: Standardized mean difference
t-test: Student’s t-test
MWU test: Mann–Whitney U test
